# Comparison of Modified Chandler, Roller Pump, and Ball Valve Circulation Models for *In Vitro* Testing in High Blood Flow Conditions: Application in Thrombogenicity Testing of Different Materials for Vascular Applications

**DOI:** 10.1155/2012/673163

**Published:** 2012-05-09

**Authors:** Wim van Oeveren, Ignace F. Tielliu, Jurgen de Hart

**Affiliations:** ^1^Haemoscan and Department of Cardiothoracic Surgery, UMCG, Haemoscan, Stavangerweg 23-23, 9723 JC, Groningen, The Netherlands; ^2^University Medical Center Groningen (UMCG), P.O. Box 9700 RB Groningen, The Netherlands; ^3^Hemolab, Den Dolech 2, 5612 AZ Eindhoven, The Netherlands

## Abstract

Three different models, a modified Chandler loop, roller pump, and a new ball valve model (Hemobile), were compared with regard to intrinsic damage of blood components and activation of platelets. The Hemobile was used for testing of polymer tubes. High flow was not possible with the Chandler loop. The roller pump and the Hemobile could be adjusted to high flow, but he pump induced hemolysis. Platelet numbers were reduced in the roller pump and Chandler loop (*P* < 0.05), but remained high in the Hemobile. Platelet aggregation was reduced in all models. The Hemobile was applied for testing vascular graft materials, and allowed different circuits circulated simultaneously at 37°C. ePTFE, Dyneema Purity UHMWPE fiber and PET fiber based tubes, all showed hemolysis below 0.2% and reduced platelet count and function. Binding of fibrin and platelets was higer on PET, inflammatory markers were lowest on Dyneema Purity UHMWPE. We concluded that the Hemobile minimally affects blood and could be adjusted to high blood flows, simulating arterial shear stress. The Hemobile was used to measure hemocompatibility of graft material and showed Dyneema Purity UHMWPE fiber in many ways more hemocompatible than ePTFE and PET.

## 1. Introduction

The use of medical devices for temporary use or implantation in the blood circulation has resulted in an increased demand for evaluation of complications brought about by these devices. This resulted also in-better defined ISO requirements for testing [1] One important and relevant aspect of testing of medical devices is the condition of blood exposed to the device. Anticoagulation and flow conditions must be as similar as possible as in the clinical setting in order to achieve relevant test results. For some devices, including grafts, stents, and catheters, this implies similar anticoagulation treatment as used in clinical situations, but also high flow through or around the device to obtain relevant shear stress conditions [2, 3]. However, in some reports, blood was treated with other anticoagulants than in clinical situations. In addition, test devices were incubated under static conditions [4–7].

Flow models for testing may consist of animal models or *in vitro* test systems. Animal models should be avoided if equally quality results may be obtained in other ways. In addition, animal blood has essential differences compared with human blood, particularly with regard to clotting and platelet function. Two types of *in vitro* flow models (often with human blood) have been used extensively: the modified Chandler loop and the roller pump closed-loop system. The modified Chandler loop [8] consists of closed tubing partly filled with air. On rotation, devices in the tubing repetitively circulate through the air-liquid interface. Use of this system may induce artefacts due to major forces applied to blood elements and protein denaturation at the air-liquid interface [9–11]. Another flow model is the roller pump closed-loop system. This model appeared effective for short circulation times [12–14], but intrinsic blood damage reduces sensitivity and does not allow prolonged exposure to blood [15]. To overcome the disadvantages of these models, the Hemobile was constructed. It consists of a simple mechanical device which generates a semicircular movement. In addition, the tubing contains no air, and there is no mechanical device compressing the tubing. In this way it was attempted to reduce damage and activation and to simulate pulsatile flow in a frequency similar to the arterial circulation. The Hemobile model was compared with the Chandler loop and roller pump model for intrinsic damage to blood components and activation of platelets. The Hemobile was further used in testing tubular structures made of various polymers to show its effectiveness in determination of hemocompatibility by means of *in vitro* circulation of human blood.

## 2. Materials and Methods

### 2.1. Blood Collection

Fresh human blood was obtained by vena puncture with a 19 Gauge needle under low pressure from five healthy adult volunteers and anticoagulated with a clinical dose of heparin (1.5 IU/mL) (Leo Pharmaceutical Products BV, Weesp, The Netherlands).

### 2.2. Before Incubation

Before starting the experiment, cell count (cell counter Medonic CA 530, Medonic, Sweden) and platelet function analysis (Platelet Function Analyzer-100) were performed and platelet aggregation was determined by adding 50 mMol ADP to a mixture of 500 *μ*L of blood and 500 *μ*L of saline and with the use of a whole blood aggregometer (Chrono Log Corp. aggregometer, Kordia Life Sciences, Leiden, The Netherlands) to confirm proper blood quality relevant for this study and to provide baseline characteristics.

### 2.3. Flow Models

**Figure 1:**
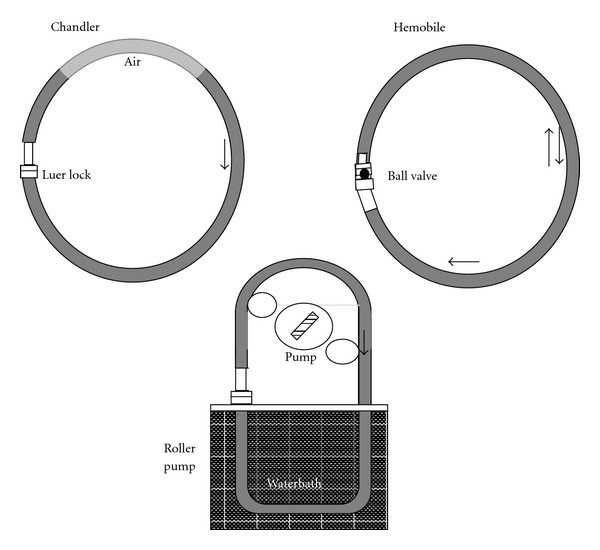
Schematic presentation of* in vitro *modified Chandler loop, Hemobile, and roller pump circulation model. All models could contain a chamber in the circulation loop, containing a biomaterial or product for testing.

Three circulation models were compared; the modified Chandler loop, roller pump, and Hemobile model (Figure 1). The principle of the modified Chandler model is based on a chamber of air that remains on top of a vertical rotating circular loop (De Spatel BV, Roden, the Netherlands). The roller pump is a nonocclusive pump for clinical use in extracorporeal circuits (Stöckert, Munich, Germany). The Hemobile (Haemoscan BV, Groningen, the Netherlands) consists of a cylinder, which is forced in a semirotating movement. On this cylinder the circular loop circuits can be positioned. Due to the semirotating movement and the slowness of blood, a pulsating movement of blood through the circuits is generated which mimics pulsatile flow. A ball valve ensures a directed flow of up to 40 mL/min through 3 mm tubing at 60 beats/min, thus creating shear stresses of 12 dynes/cm^2^. Also, the flow wave form generated by the model is more physiological compared to the roller pump and Chandler model.

### 2.4. Study Design Model Validation

A comparative study was performed to assess intrinsic blood damage for three different closed-loop circulation models, without any test device in the circuit. Each set of experiments (three different circuits in triplicate) was performed five times with fresh venous blood from a different donor for each set. Due to restrictions of the Chandler loop model, the flow through 3 mm tubing was limited to 25 mL/min (6 dynes/cm^2^) in all experiments. The first set of experiments was performed under circumstances of similar flow (25 mL/min) in all three test systems. 

The following sets of experiments (*n* = 4) were performed with the roller pump and Hemobile at an exceeded blood flow of 40 mL/min (12 dynes/cm^2^), which resembles shear stresses in the coronary blood circulation. 

After these validation experiments, the Hemobile was used to test the hemocompatibility of woven tubular structures from polyester (PET) fiber and Dyneema Purity UHMWPE fiber, in comparison with expanded polytetrafluoroethylene (ePTFE) tubes. Platelet function, thrombosis, and hematology were tested according ISO10993/4.

### 2.5. Incubation

All experiments were performed with closed PVC tubing circuits (Raumedic) with a length of 45 cm and an internal diameter of 3 mm. The Chandler loop and roller pump circuits contained Luer-lock connectors. The Hemobile circuits were fitted with a ball valve connector (Halkey-Roberts, Street Petersburg, FL, USA). The roller pump circuit was partially immersed in water, whereas the Chandler loop and Hemobile were completely placed in an oven (Figure 1). The temperature in all three circuits was permanently kept at 37°C. The circuits of the roller pump and Hemobile were filled with 4.5 mL of blood and the circuits rotating on the Chandler loop system with 4.0 mL of blood and 0.5 mL of air.

### 2.6. After Incubation

After one hour of circulation, the circuits were emptied. Cell count, platelet function analysis, and platelet aggregation measurements were performed on the circulated blood, as described above. The rest of the blood was mixed with 0.2 M EDTA and centrifuged at 11,000 ×g for one minute. Plasma was used for analysis of thromboxane B2 (TXB2), Thrombin-Antithrombin III (TAT) complexes, elastase, and hemoglobin concentrations.

Activation of the arachidonic acid pathway in platelets results in the release of the potent platelet aggregating agent thromboxane A2, which is rapidly converted to the inactive product TXB2. TXB2 was measured by means of an enzyme immunoassay (Biotrak, Amersham, UK), based on competition of labelled TXB2 with sample TXB2. In this test the label is a peroxidase, which converts the substrate tetramethylbenzidine, yielding a yellow colour which is measured at 450 nm by a spectrophotometer (Powerwave 200, Biotek Instruments, Winooski, VT, USA).

TAT was measured as an indication of coagulation activity. Thrombin formation during the *in vitro* experiments was determined by means of TAT complexes in EDTA plasma. A TAT ELISA was performed with capture and detection of antibodies from Cederlane Laboratories (Hornby, Canada).

Release of polymorphonuclear (PMN) elastase *in vivo* is a specific marker for inflammation reactions. Free in plasma, elastase is rapidly neutralised by *α*1-antitrypsin inhibitor to form a stable complex. Elastase was determined by ELISA by means of capture antibody against human elastase and labelled detection antibody against alpha1 antitrypsin (Affinity Biologicals, Ontario, Canada).

Complement activation was determined by ELISA based on a mouse anti human C5-9 antibody (DAKO, Glostrup, Denmark) and goat anti-C5 detection antibody (Quidel, San Diego, CA, USA).

Free hemoglobin as an index for erythrocyte damage was measured as described by Harboe [16] and using a spectrophotometer (Power Wave 200). The emptied PVC circuits and the incubated graft material were washed with Tris-buffered saline (pH 7.4). Platelet adhesion onto the surface of the circuits was measured by means of a colorimetric assay, based on the presence of acid phosphatase in platelets [17]. Platelet binding was measured based on the release of platelet acid phosphatase in Citrate buffer (pH 5.4), containing p-nitrophenyl phosphatase and Triton X100. Substrate conversion is proportional to the amount of platelets, which was determined by a standard curve and platelet counting.

Scanning electron microscopy (SEM) was achieved on material fixated in 2% glutaraldehyde in cacodylate buffer, treated with osmium tetraoxide in cacodylate buffer and gold sputtered critical point dried samples. These were visualized at 2 kV (Jeol 6301 F, Jeol Ltd., Tokyo, Japan).

### 2.7. Statistical Analysis

Normally distributed variables were reported as mean with standard deviation (SD). ANOVA was performed for all blood parameters to assess any difference in blood cell damage or activation between the circuits. Posthoc evaluation was performed by *t*-test.

## 3. Results

### 3.1. Whole Blood Assays at 25 mL/min Flow

**Figure 2:**
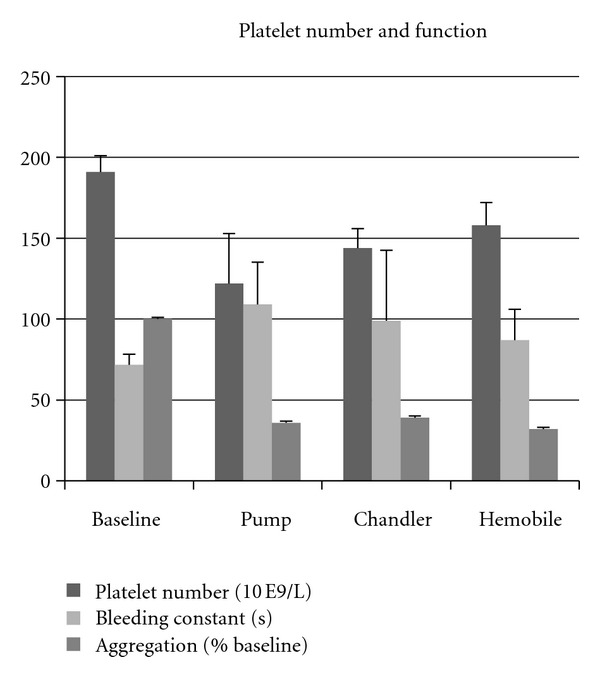
Effects of three circulation models on platelets. Platelet number declined in all models, while bleeding time increased and aggregation reduced.

Platelet-count was significantly reduced by the roller pump and by the modified Chandler loop, but not by the Hemobile (Figure 2). Platelet function was partially decreased in all systems, in particular in the roller pump system. Platelet adhesion to the PVC tubing was lowest in the Chandler loop (Table 1).

### 3.2. Plasma Assays

TXB2 and free hemoglobin were significantly higher after roller pump circulation than after Chandler loop and Hemobile circulation (Table 1). TAT was similarly increased in all systems.

### 3.3. Results at 40 mL/min Flow

Pump and Hemobile could be applied at a flow of 40 mL/min, which was not possible with the Chandler model. Platelet number remained higher, and hemolysis was lower in the Hemobile circuits (Table 2).

### 3.4. Application of the Hemobile in Thrombogenicity Testing

**Figure 3:**
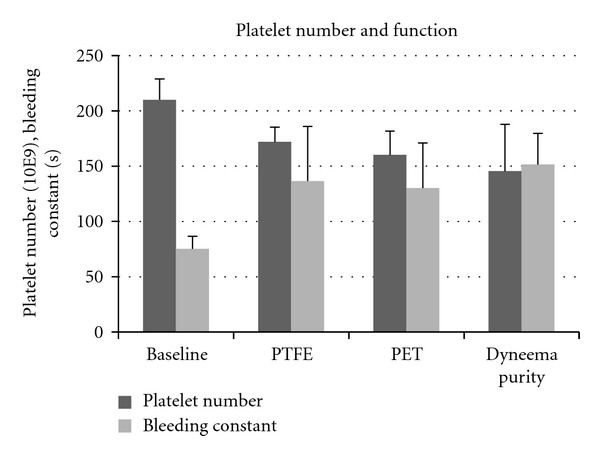
Effect of three types of graft on platelet function. Platelet number and functions were similar after contact with vascular graft materials made of ePTFE, PET or Dyneema Purity UHMWPE fiber.

**Figure 4:**
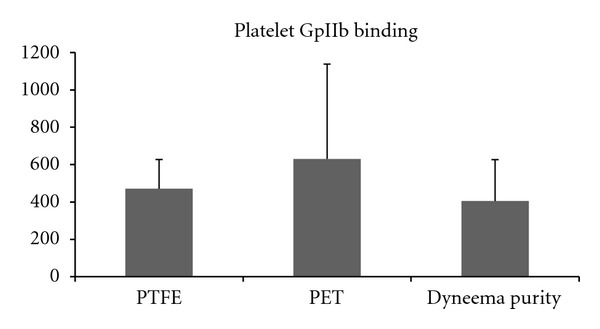
Effect of three types of graft on platelet binding (receptor GpIIb). Platelet adhesion was lowest in the vascular grafts made of Dyneema Purity UHMWPE fiber, as compared to ePTFE and PET.

Platelet count was reduced in the circuits containing a test chamber with ePTFE, PET, or Dyneema Purity UHMWPE fiber by approximately 30% following circulation compared to baseline. Platelet function was reduced in all circuits to a similar extent (Figure 3). Platelet binding based on the adhesion of antibody against the platelet GpIIb receptor showed increased values on PET, whereas Dyneema Purity UHMWPE fiber had lowest GpIIb receptors (Figure 4). In the ePTFE circuits, release of TXB2 and TAT was lowest, whereas elastase, complement activation, and hemolysis were lowest in Dyneema Purity UHMWPE fiber circuits (Table 3). 

### 3.5. Scanning Electron Microscopy

**Figure 5:**
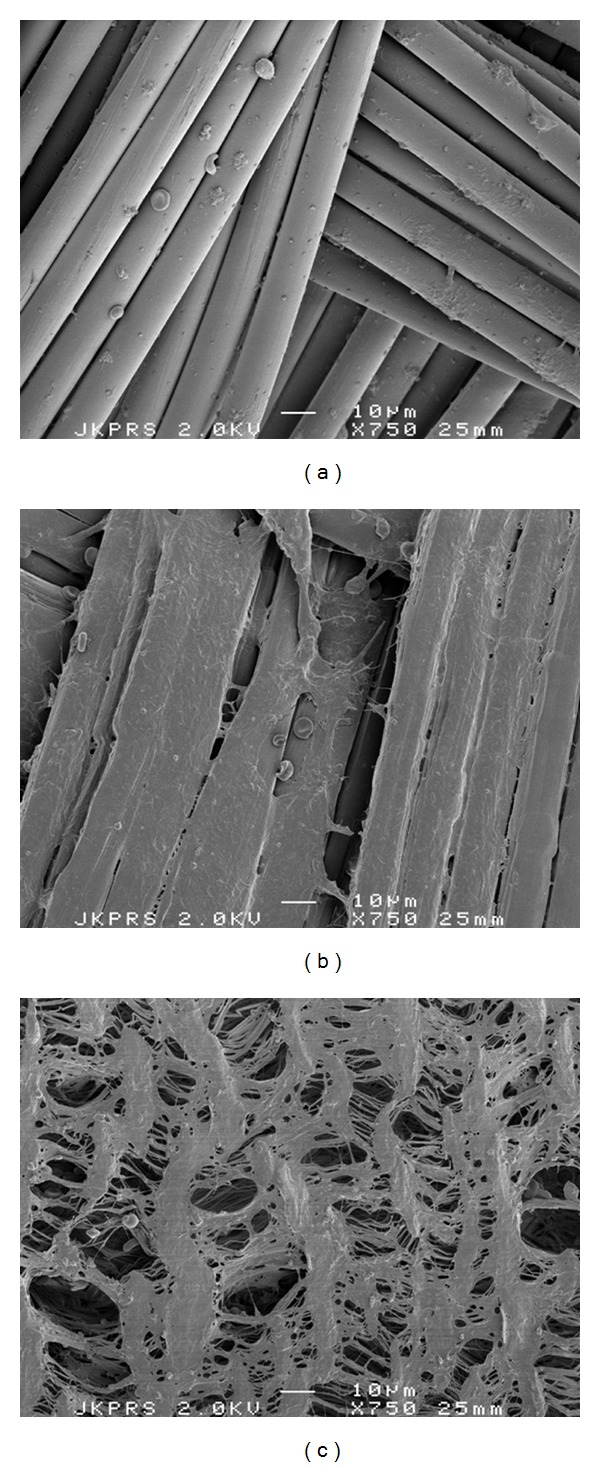
Scanning Electron Microscopy. (a) PET fiber: platelets clusters and erythrocytes were observed attached to the fibers. (b) Dyneema Purity UHMWPE fiber weave: separate platelets some erythrocytes and a protein layer were observed on the fibers. (c) Expanded PTFE: small clusters of platelet were observed.

SEM photos were made of all types of vascular graft materials. More detailed pictures showed platelets and fibrin usually separate and not as a dense thrombus on the surface. Differences between the 3 types of graft materials were not clear from these pictures, although ePTFE seemed to remain more devoid of deposition than PET and Dyneema Purity UHMWPE fiber (Figure 5).

## 4. Discussion

Initial experimental blood circulation models with a roller pump, already refined in previous studies [13, 14, 18], appeared efficient, reliable, and cost-effective to assess the haemocompatibility of grafts before their clinical use. However, blood damage induced by the pump caused a limitation of the exposure of the test object to circulating blood. The modified Chandler loop model is currently most frequently used for these purposes [19, 20]. It induces less blood damage than the roller pump but has two major disadvantages. First, the continuous blood-air contact induces leukocyte and platelet aggregation, protein denaturation [9–11], and shear forces on particulate material, which can result in detachment [21, 22]. In our experiments, indeed less platelets were observed on tubing exposed to blood-air contact in the Chandler loop than in the roller pump or Hemobile circuit. The second disadvantage of the Chandler loop is the limitation of blood circulation due to the requirement to keep the air on top of the circuit. Air tends to circulate with the tubing at higher circulation speed. Thus, our 3 mm tubing did not allow a blood flow over 25 mL/min, which is half of the arterial flow in the coronary system. At a flow of 25 and 40 mL/min, the Hemobile could be used and was less traumatic for blood than the roller pump model.

It is well known that any type of foreign body material can be thrombogenic by promoting the formation of thrombin and platelet activation, which facilitates platelets to adhere and to express surface receptors (Gp IIbIIIa) of activated phase [23]. Platelet binding becomes then irreversible and can promote more thrombus formation. Therefore, proper testing of the characteristics of graft material is an important issue in modulating blood interaction. The small tubular system used in the present models allows multiple tests with fresh human blood. The modified Chandler loop and Hemobile can be easily loaded with a number of circuits at a time. Fast screening of thrombogenicity of vascular grafts and other small medical devices is possible. The adjustable flow and shear and the pulsatility in the Hemobile renders it in a model that allows standardised testing of these devices at the cost of low intrinsic blood damage, while closely mimicking the *in vivo* conditions. Our results indicated that the changes in circulating blood are most of all dependent on the material used in the test loop. Moreover a direct comparison of material surfaces was possible by using blood of the same donor for different circuits.

The feasibility of the Hemobile has been demonstrated by applying the model in hemocompatibility studies of different vascular graft materials. 

Woven tubes made of PET fiber and Dyneema Purity UHMWPE fiber have been compared to commercially available ePTFE vascular graft. Our results showed that Dyneema Purity UHMWPE fiber has in many ways better properties than ePTFE by lower activation of the inflammatory response and lower hemolysis.

A limitation of *in vitro* models is mainly represented by the absence of an endothelial layer in the circulating system. Throughout the release of cytokines, the endothelium has a major role in mediating the interplay between the injured vessel wall and circulating blood cells [24–26]. This is effectuated by the release of cytokines and (anti)thrombotic components as well as the expression of adhesion molecules. The lacking of this character can somehow alter the likelihood of our experimental representation. Prior to use in patients an animal model should prove the validity of the *in vitro* data. Nevertheless, all the other elements depicting the blood-graft phase boundary scene are present, while the use of human blood from one donor in test and control circuits is a major advantage for comparison of the materials.

## Figures and Tables

**Table 1:** Activation markers in plasma after Chandler loop, roller pump and Hemobile blood circulation at 25 mL/min.

	Baseline	Chandler	Roller pump	Hemobile
TxB2 (pg/mL)	34 ± 12	712 ± 296	1222*±166	879 ± 167
TAT (*μ*g/mL)	3.2 ± 1.7	66.7 ± 21.5	65.4 ± 24.4	64.3 ± 20.6
Hemolysis (gHb/L)	0.07 ± 0.06	0.21 ± 0.19	0.85** ± 0.14	0.21 ± 0.06
Platelet adhesion	—	0.46^#^ ± 0.41	0.72 ± 0.42	0.89 ± 0.53

*P < 0.05, **P < 0.01 compared to Chandler loop and Hemobile; ^#^ *P* < 0.05 compared to roller pump and Hemobile

TXB2: tromboxane B2; TAT: thrombin-antithrombin III.

**Table 2:** Roller pump and Hemobile performed at 40 mL/min blood circulation.

	Baseline	Roller pump	Hemobile
Platelet number (10E9/L)	115 ± 36	70 ± 28	117 ± 32*
Bleeding constant (sec)	72 ± 7	108 ± 20	99 ± 21
Aggregation (slope)	18.7 ± 7.1	6.9 ± 3	8.7 ± 4.7
Adhesion (10E6/cm^2^)	—	0.75 ± 0.73	0.53 ± 0.45
Hemolysis (gHb/L)	0.07 ± 0.06	0.95 ± 0.51	0.18 ± 0.11**

*P < 0.05; **P < 0.01 compared to roller pump.

**Table 3:** Activation markers in plasma after Hemobile circulation at 40 mL/min in the presence of different grafts (average ± SD).

	Baseline	ePTFE	PET	Dyneema Purity
TxB2 (pg/mL)	21 ± 14	779 ± 642	964 ± 428	948 ± 246
TAT (*μ*g/mL)	0.7 ± 0.7	73.7 ± 118	147.6 ± 53	90.0 ± 131
elastase (*μ*g/mL)	0.32 ± 0.35	4.36 ± 5.27	3.00 ± 3.01	2.85 ± 2.97
C5b-9 (U/mL)	1.55 ± 2.17	3.31 ± 3.01	3.41 ± 2.82	2.60 ± 2.63
hemolysis (gHb/L)	0,02 ± 0.01	0,14 ± 0.1	0,15 ± 0.11	0,08 ± 0.002

TXB2: thromboxane B2; TAT: thrombin-antithrombin III.
